# Engineering Application of Hardbanding Technology in the Petroleum Industry

**DOI:** 10.3390/ma17246075

**Published:** 2024-12-12

**Authors:** Marius Bădicioiu, Mihaela Mădălina Călțaru, Marius Gabriel Petrescu

**Affiliations:** Mechanical Engineering Department, Petroleum-Gas University of Ploiești, 100680 Ploiesti, Romania; mbadicioiu@upg-ploiesti.ro (M.B.); pmarius@upg-ploiesti.ro (M.G.P.)

**Keywords:** NC50 tool joints, reconditioning, ARNCO 100XT and FLUXOFIL M58 wear-resistant materials, GMAW process, welding procedure specifications, quality control

## Abstract

The petroleum industry is essential for supplying crude oil, which is vital for fuel and chemicals and drives substantial investments in technologies, especially in regard to increasing the durability of the drill strings used in wellbore construction. This study aims to establish and to validate a hardbanding technology for reconditioning NC50 tool joints subjected to wear, thereby increasing drill pipe durability and reducing the risk of failure during drilling, which can lead to ecological pollution, human safety issues, and financial costs. The hardbanding of the tool joints was carried out using the gas metal arc welding process (GMAW) with two different wear-resistant wires, ARNCO 100XT and FLUXOFIL M58. The equipment designed for this research allowed for the application of high-quality hardbanding layers in very good condition, according to the favorable results obtained by macroscopic analyses, metallographic studies and hardness measurements. The hardbanding procedure of the tool joint drill pipe was documented in a welding procedure specification (WPS), which validates the process and is useful for operators, drilling companies and other oilfield service companies who wish to apply under the same conditions and at the same high-quality level, in a repetitive mode, the reconditioning process to minimize the risk of drill-string failures.

## 1. Introduction

The petroleum industry constitutes one of the most important and complex industries in the world because it provides the raw material (crude oil) necessary for the preparation of fuels such as oil, gasoline and other chemical products (pharmaceuticals, plastics, pesticide and lubricants); it is also one of the industries in which massive investments are made to create high-performance technologies and equipment. This industry encompasses worldwide activities regarding the exploration, extraction, refining and transportation of petroleum products.

**Figure 1:**
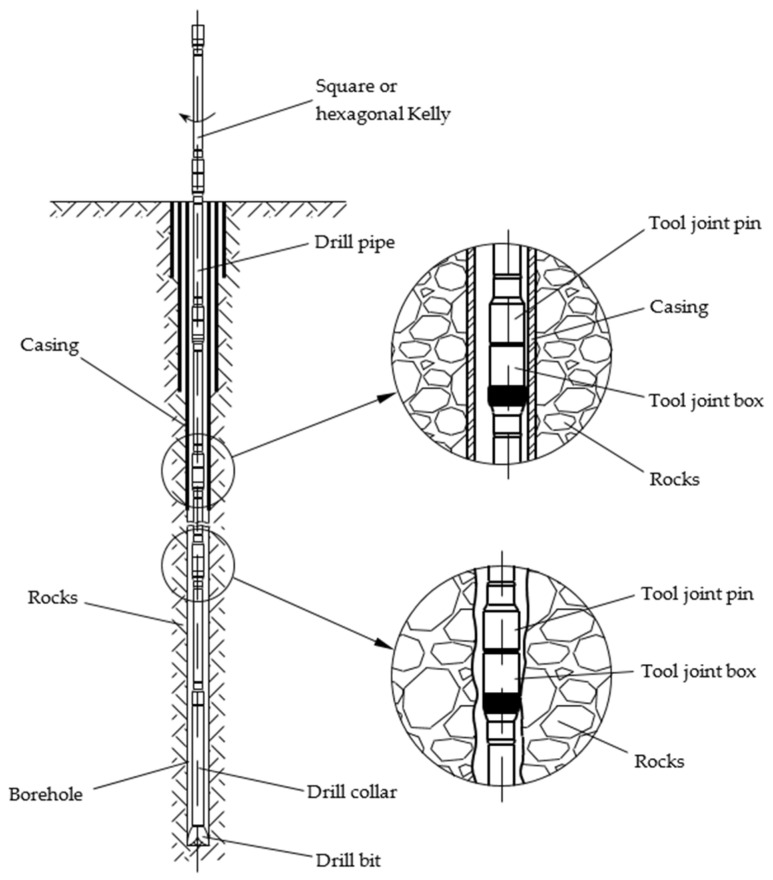
Schematically representation of the drill pipe.

To extract crude oil, it is necessary to drill a borehole to the depth of the raw material. This process is performed with the help of a drill string, at the base of which the drill bit is mounted with the aim of dislocating the rock and forming the borehole. The drill string consists of several key components [1,2,3,4]. First, there are the drill pipes, which play a crucial role in transmitting the rotary motion from the Kelly to the drill bit while also carrying the drilling fluid to the bit. Additionally, there are drill collars, which are essential for maintaining the drill pipes in tension and providing the necessary weight on the bit for effective drilling. Finally, the square or hexagonal Kelly is responsible for transmitting torque from the rotary table to the entire drill string (Figure 1). During the drilling process, the casing strings are used to ensure the integrity of the borehole in each drilling area.

**Figure 2:**
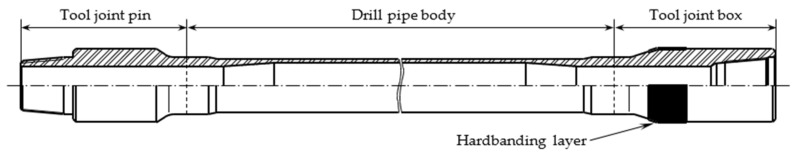
Drill pipe schematic representation.

The drill string is subjected to a complex stress state due to tensile–compressive loading, torsional loading, bending loading, and internal and external pressure in very severe working environmental conditions, factors that may lead to accidental failures during the drilling process [1,2]. One of the forms of degradation frequently encountered at the drill string is the wear of the drill pipes’ outer surfaces. The drill pipes consist of three distinct components welded together: the drill pipe body (thick-walled pipe with thickened ends), the tool joint pin and the tool joint box (Figure 2) [3].

The wear degradation of the drill pipes is located, in particular, in the area of the tool joint box connection. This phenomenon is largely present due to the drill pipe tool joint friction with the geological formations (rocks) of the borehole or with the inner walls of the casing string [5,6,7,8]. In the manufacturing process of the drill pipes, the tool joints are covered with a wear-resistance material, applied in circumferential layers (hardbanding layers), concentric with and normal to the tool joint axis (Figure 2), to protect them against abrasive wear. During the exploitation process, abrasive wear may occur due to friction and the hardbanding layers are partially removed, compromising the drill pipes’ integrity, which may affect the structural integrity of the well, resulting in serious negative consequences to the environment (contamination), human security and financial expenses. Thus, to prevent any accidents by failure of the drill pipes, the tool joints are reconditioned by hardbanding in order to meet the originally designed quality requirements for working in safety conditions.

“Hardbanding” is a surface-coating process where a layer of hard material is deposited on the surface of a metal part, usually made from a softer material. The deposition of the hard material can be achieved through various technological processes that ensure adhesion between the materials involved in the hardbanding process. The welding technological process is used for hardbanding when the deposit has to be very dense and thick, with good adhesion and a high-strength bond [9]. Research in the field of surface engineering processes is focused on developing new technologies and new materials for performance improvement, profitability, and environmental protection [10]. The hardbanding technologies are applied all over the world in different sectors (the manufacturing industry, chemical industry and petroleum industry) to extend the service life of the parts subjected to wear in the exploitation process. Therefore, numerous researchers have analyzed the conditions and the effects of the hardbanding process applied to the reconditioning of different machine and equipment parts. In [11], the authors present a comparison of reconditioning processes for crankshafts through welding using shielded metal arc welding (SMAW) and wolfram inert gas welding (WIG), focusing on the metallographic structures obtained in the areas resulting from the hardbanding process. The authors conclude that reconditioning by welding offers the possibility of an effective control of the heat input in the reconditioned parts, which guarantees obtaining metallographic microstructures with a lower occurrence of defects during the exploitation process. A lower heat input minimizes the thermal damage, dilution and distortion of the parent metal and a higher heat input increases the area with lower hardness, according to Durga Tandon et al. [9]. Minh-Tan Nguyen et al. [12] studied the hardness and wear resistance characteristics of the hardbanding layers deposited through electric resistance seam welding on the surface of deteriorated shafts. Their studies included nine different technologies (welding conditions) where steel wire was used as filler metal. Rajeev Ranjan [13] noted that hardbanding performed using the gas metal arc welding (GMAW) process significantly improved the surface wear resistance of the part. This finding was based on a study that examined the influence of welding process parameters on the characteristics of hardbanding layers. According to this study, the optimal properties of the hardbanding layers (bead width, height of the bead, penetration, mechanical strength and wear and corrosion resistance) can be assured by a proper surface preparation of the part and by using the adequate technological parameters (arc voltage, welding current, travel speed, stand-off distance and welding gun angle). In the petroleum industry, the hardbanding process is commonly used to protect the drill string from wear and to prevent casing wear [14,15,16,17,18,19]. The article [20] highlights an application developed by Arnco Technology for reconditioning drilling components through hardbanding with a chromium carbide-based alloy, Arnco 200XT. The process demonstrated excellent results, significantly reducing wear as well as forces and torque during drilling. In the same context, it is important to mention the interest of various companies in developing their own hardbanding materials and technologies for reconditioning or even improving the quality of tools and equipment, particularly in the drilling industry. An example is the cooperation between Castolin Eutectic and Statoil [21], which resulted in the development of new, innovative hardbanding materials and the patent of specific reconditioning technologies. Thus, research regarding the development of reconditioning materials and technologies cannot be concluded without validation of the specific technological procedures. After quality control analyses of the hardbanding, the procedure must be documented in a welding procedure specification (WPS). The WPS document validates the process and is mandatory for the hardbanding procedure’s implementation, allowing companies to perform, in repetitive mode, the process under the same conditions at the same high-quality level. This conclusion is in line with the present article, which presents not only the results of research regarding the reconditioning process by hardbanding but also the validation of the specific procedure.

The main aim of the present study is to establish, by experimental research, the gas metal arc welding procedure specifications (WPSs) applied for reconditioning of the NC50 tool joint box subjected to wear by hardbanding using two different wear-resistant materials named ARNCO 100X and FLUXOFIL M58, respectively. The methodology developed, the equipment designed and the results obtained during this research certify the quality of the hardbanding process necessary for operators, drilling companies or other oilfield services interested in improving the durability of the drill pipes by hardbanding the tool joints subjected to wear.

## 2. Materials and Methods

### 2.1. Materials

**Figure 3:**
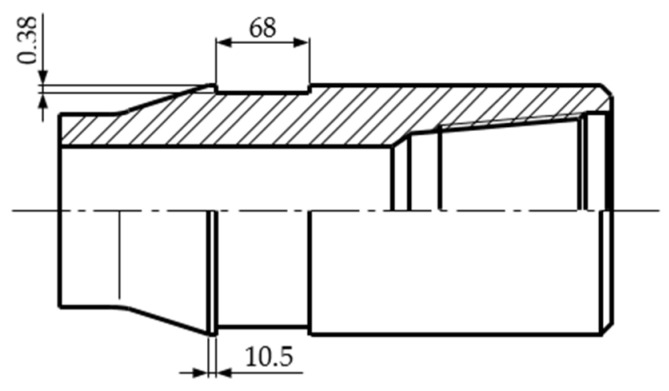
A machined recess grove machined on the tool joint surface.

The experimental research, in laboratory conditions, was performed on an NC50 used tool joint box with a 6 5/8 outer diameter, manufactured by steel grade 38CD4 (0.15…0.25% Mo, 0.6…0.9% Mn, 0.9…1.2% Cr, 0.35…0.41% C) [22]. The tool joints were hardbanded by the manufacturer of the drill pipes. The hardbanded surfaces of the tool joints were subjected to wear during the exploitation process in the wellbore. To prevent drill-string failure, the used tool joints were subjected to a reconditioning process by applying new hardbanding layers onto the external surface of the tool joint, respectively, in the machined recess grove, according to Figure 3. During the machining process, the hardbanded material subjected to wear (old hardbanding layers), applied by the manufacturer, was partially removed.

In order to recondition the NC50 tool joints by using the GMAW process, two different wear-resistant materials were used as filler materials. The filler material had to be suitable for the GMAW process, to avoid the oxidation reaction due to the thermal dissociation of the carbon dioxide (CO_2_) used as shielding gas and, also, to ensure the achievement of tool joint hardbanded layer characteristics suitable for exploitation conditions and minimizing wear on the casing in the borehole. A Rockwell hardness (HRC) of tool joint hardbanded layers higher than 57–62 HRC will cause an excessive wear of the casing due to the friction process [1,2,23,24].

Based on these aspects, in experimental works, ARNCO 100XT and FLUXOFIL M58, were used as two different wear-resistant flux core tubular wires of 1.6 mm diameter, having the characteristics, according to manufactures, presented in Table 1 [23,24,25]. Both tubular wires are suitable for hardbanding with the GMAW process of reconditioning wear parts subjected to heavy wear.

The high values of hardness for the hardbanded layers, based on the chemical compositions presented in Table 1, are attributed to the chromium and molybdenum carbides formed in the metal base that can be made bainitic due to the high carbon content.

The shielding gasses used for NC50 tool joint reconditioning by using the GMAW process were 82% Ar and 18% CO_2_ (in the case of hardbanding with FLUXOFIL M58), respectively, as well as 100% CO_2_ (in the case of hardbanding with ARNCO 100 XT).

### 2.2. Hardbanding Equipment

**Figure 4:**
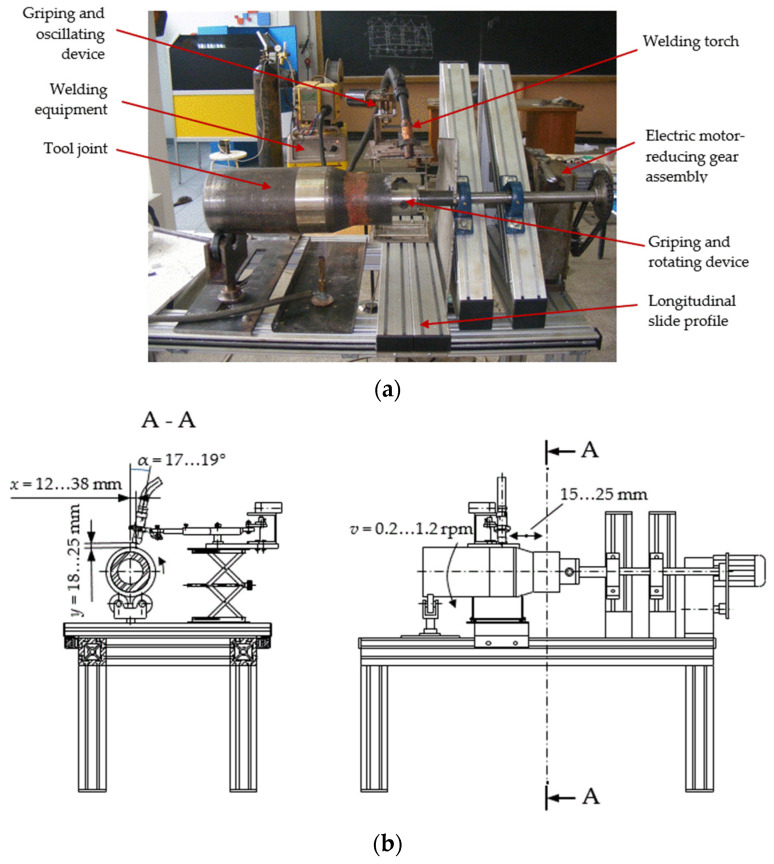
Hardbanding equipment: (**a**) Image of the hardbanding stand in the Welding Laboratory from the Petroleum-Gas University of Ploiesti; (**b**) schematic representation of the hardbanding stand [25].

In order to perform the experimental research, the hardbanding stand presented in Figure 4 was designed and manufactured to allow for the following movements [24]: continuous rotation of the tool joints, axial feed of the welding torch and oscillation of the welding torch.

The tool joints were fixed in the griping and rotating device (a three-jaw self-centering lathe chuck), which ensured their correct centering.

The hardbanding stand was designed to ensure different and constant rotating speeds in the tool joints, according to their outer diameter, ranging from 0.2 to 1.2 rotations per minute using an electric motor–reducing gear assembly.

The axial feed of the welding torch was realized with the longitudinal slide profile that supports the griping and oscillating device of the welding torch.

To ensure the oscillating movement of the welding torch, a device with an eccentric articulated lever, operated by a direct current (DC) gear motor (the oscillating speed at ranging approximatively 60 to 90 oscillations per minute, and an amplitude of 15 to 25 mm) was used.

**Figure 5:**
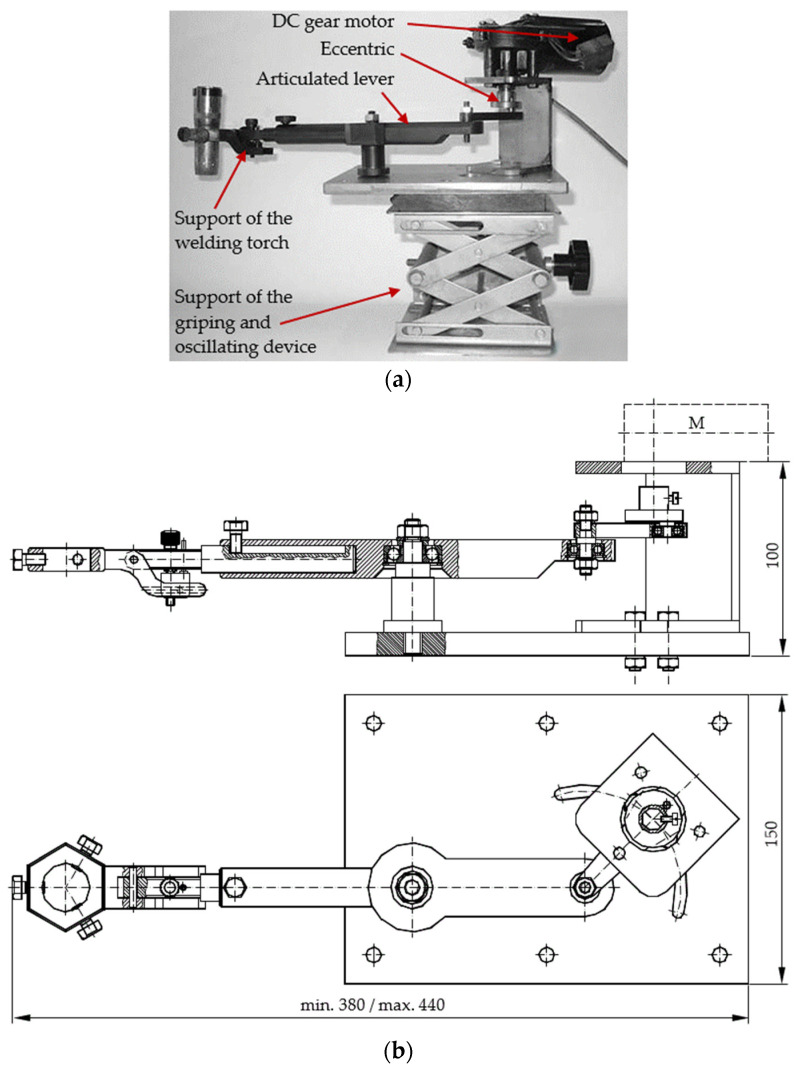
Griping and oscillating device: (**a**) image of the griping and oscillating device manufactured in the laboratory fat the Petroleum-Gas University of Ploiesti; (**b**) assembly drawing of the gripping and oscillating device designed by the authors of this article [25].

In addition, the griping and oscillating device allows for tilting the welding torch to an angle between 17 and 19 degrees, as measured from the centerline of the tool joint. The oscillating and the griping device of the welding torch is present in Figure 5.

The welding equipment, using the GMAW process, must have a constant direct current power supply capable of providing a current of 180 to 400 Amperes and 20 to 30 Volts with positive wire (reverse) polarity. The welding equipment type ARISTO LUD 450 was utilized for hardbanding the tool joint using the GMAW process in laboratory conditions.

### 2.3. Hardbanding Methodology

#### 2.3.1. Tool Joints Preparation

After turning the tool joints using a PROMA SPF-1000P lathe (manufactured by PROMA Machinery & Tools, Prague, Czech Republic), in order to machine the recess grove (Figure 3), a visual inspection of the tool joint surfaces was performed to ensure that they were clean and free of rust, grease or other impurities.

#### 2.3.2. Preheating the Tool Joints

Before the hardbanding process, the tool joints were preheated at 150 °C by using a methane gas burner. During the preheating time, the temperature was measured using a contact thermocouple. Due to the rotating of the tool joint during the preheating process, the distribution of the temperature was uniform across the entire circumference of the specimen.

#### 2.3.3. Hardbanding the Tool Joints

The tool joints were hardbanding with the wear-resistant materials FLUXOFIL M58 and ARNCO 100XT using the GMAW process. Taking into consideration the specialized literature regarding the surface-coating and welding processes, the wear-resistant wire manufacturer’s recommendations, and based on the authors’ background experiences, tests were performed in laboratory conditions by using different values for the hardbanding process in order to establish adequate technology and suitable parameters for tool joint reconditioning by welding [23,24,25,26,27,28]. The optimum technological parameters used for hardbanding of the tool joins under laboratory conditions are presented in Table 2.

**Figure 6:**
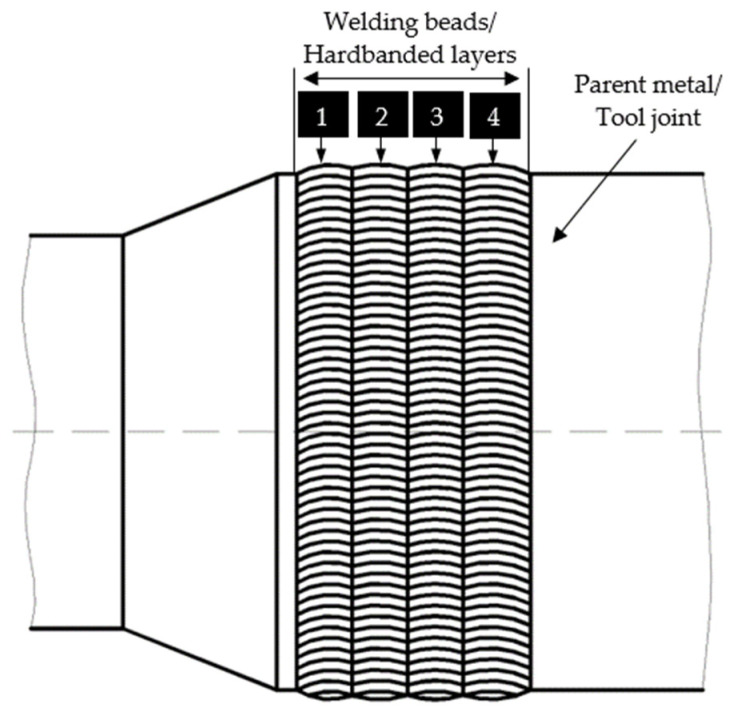
Application of the hardbanded layers.

The tool joints were hardbanded by applying four welding beads/hardbanded layers according to the order 1, 2, 3, 4, as presented in Figure 6.

**Figure 7:**
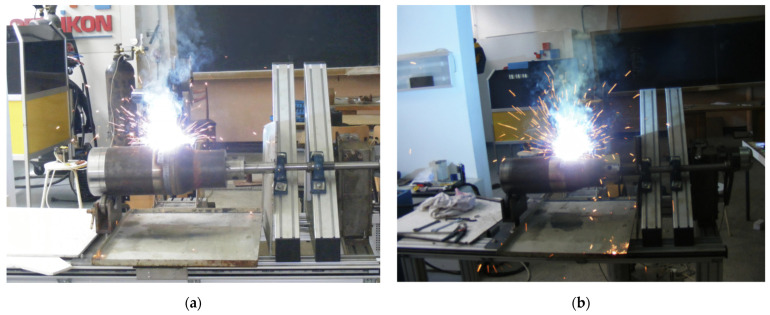
Hardbanding process: (**a**) image during hardbanding with FLUXOFIL M58; (**b**) image during hardbanding with ARNCO 100XT.

Images during the hardbanding process of the tool joints, performed in laboratory conditions, are presented in Figure 7.

#### 2.3.4. Controlled Cooling of the Hardbanded Tool Joints

**Figure 8:**
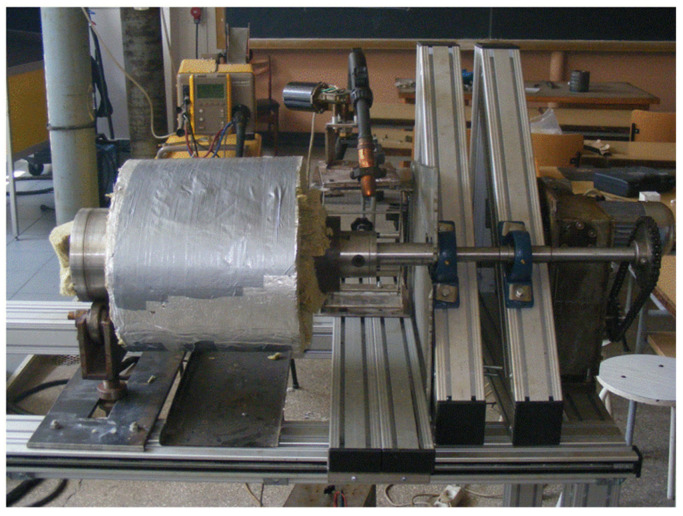
Controlled cooling of the tool joint after hardbanding.

To prevent the formation of cracks in the hardbanded layers, after finishing the hardbanding process, the tool joints were slowly cool by using a thermally insulated blanket with a thickness of 100 mm (Figure 8). In order to ensure slow cooling (with an approximatively cooling rate of 30 °C/h), and for positive results regarding the structure integrity and the hardness, the hardbanded tool joints were wrapped immediately in thermally insulated blankets. A thermally insulated blanket was used to completely cover both the exterior surface of the tool joints in the hardbanded area and the inner surface of the tool joint to prevent the any air flowing through it. During the cooling process, the temperature was monitored using a contact thermocouple until the tool joints cooled down to ambient temperature.

#### 2.3.5. Quality Control of the Reconditioned Tool Joints Hardbanded by Welding

**Figure 9:**
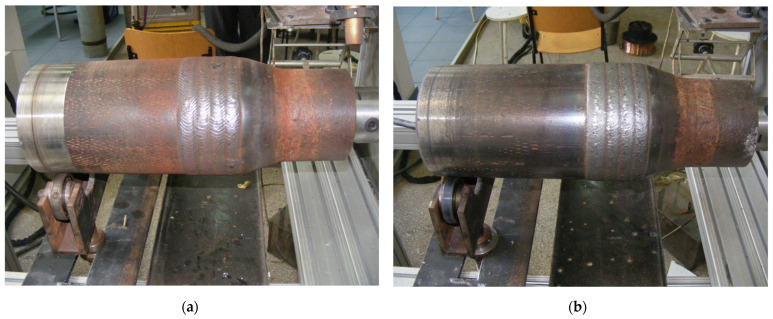
Tool joints reconditioned using the GMAW process: (**a**) image of a tool joint hardbanded with FLUXOFIL M58; (**b**) image of tool joint hardbanded with ARNCO 100XT.

The reconditioned tool joints hardbanded using the welding process, presented in Figure 9, were investigated after the cooling process in order to verify the quality of deposition.

## 3. Results and Discussion

### 3.1. Quality Control of the Hardbanded Tool Joints

**Figure 10:**
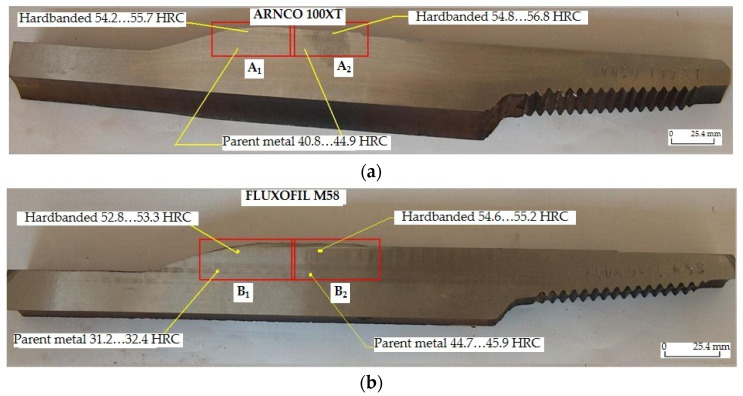
Sampling locations marked on the reconditioned tool joints and Rockwell hardness (HRC) values: (**a**) sampling location A1, A2 for tool joints hardbanded with ARNCO 100XT; (**b**) sampling location B1, B2 for tool joints hardbanded with FLUXOFIL M58.

**Figure 11:**
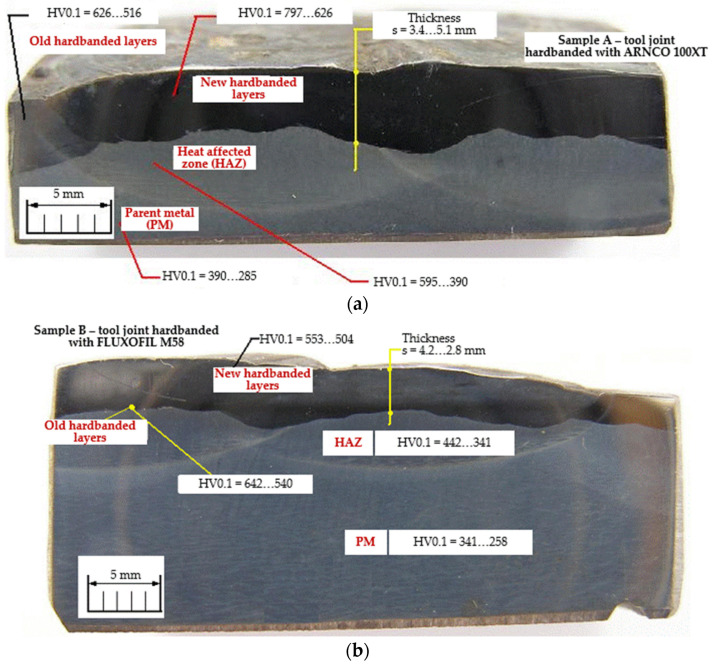
Macrophotography of the samples, hardbanding thickness(es) and Vickers micro-hardness (HV_0.1_) values: (**a**) sample A cut from the tool joints hardbanded with ARNCO 100XT; (**b**) sample B cut from the tool joints hardbanded with FLUXOFIL M58.

In order to investigate the quality of the tool joints that were reconditioned by hardbanding, samples A and B were cut from different sampling locations marked A1, A2, B1 and B2 (Figure 10) as follows:

- Sample A was cut from the reconditioned tool joints hardbanded by using the ARNCO 100XT wear-resistant tubular wire (Figure 11a);
- Sample B was cut from the reconditioned tool joints hardbanded by using the FLUXOFIL M58 wear-resistant tubular wire (Figure 11b).

Metallographic samples A and B were prepared by polishing and metallographic etching with reactive NITAL.

The quality of the hardbanded layers was investigated by performing a macroscopic analysis and a metallographic analysis using optical microscopy and hardness measurements.

#### 3.1.1. Macroscopic Analysis

The macroscopic analysis was performed by visual inspection of the hardbanded tool joints and the metallographic samples A, B; the following aspects were observed:

- The profile of the hardbanding layers are flat or slightly convex, and no cracks visible to visual inspection appeared in the deposited layers in any hardbanded tool joints;
- The adhesion between the parent metal and the new hardbanding layers, between the old and the new hardbanding layers, also between two adjacent layers is good and no voids or discontinuities were observed;
- The geometric parameters of the new hardbanding layers of the reconditioned tool joints (overall width of hardbanding 76 ± 2 mm, thickness of hardbanding layers 2.8…5.1 mm, average width of one hardbanding layer 22 mm, overlap between two adjacent hardbanding layers 4 ± 0.5 mm) is similar with the hardbanding layers of the tool joints that were not subjected to the wear process.

#### 3.1.2. Metallographic Analysis by Optical Microscopy

**Figure 12:**
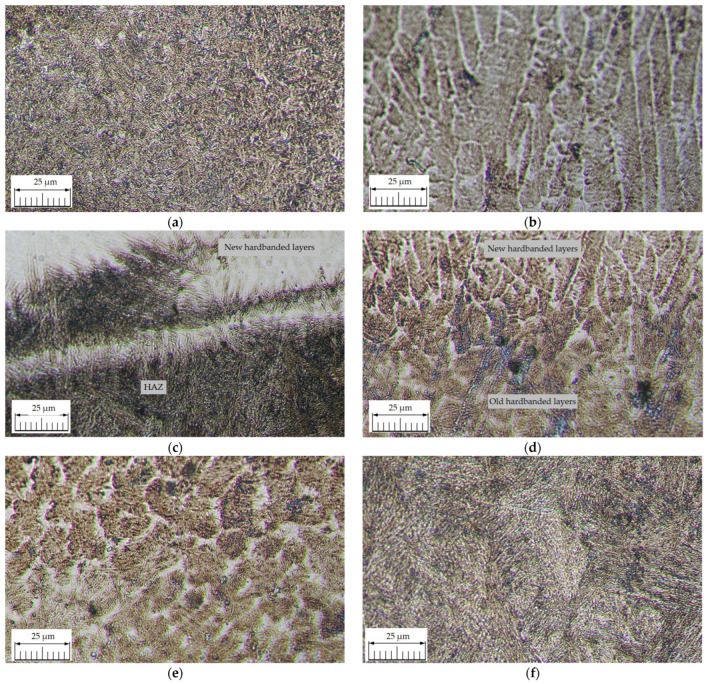
Images of metallographic structures for the reconditioned tool joints hardbanded by using the wear-resistant material ARNCO 100XT performed on sample A: (**a**) parent metal (HV0.1 = 390…285); (**b**) new hardbanding layers (HV0.1 = 797…626); (**c**) transition area between new hardbanding layers and the parent metal; (**d**) transition area between new and old hardbanding layers; (**e**) complete melting area of the old hardbanding layers (HV0.1 = 626…516); (**f**) heat-affected zone—HAZ (HV0.1 = 595…390).

The optical microscopy analysis was performed on sample A and sample B with a metallographic OLYMPUS BX60M microscope. Metallographic images of the reconditioned tool joints hardbanded with ARNCO 100XT (sample A) are shown in Figure 12.

The metallographic investigations of the reconditioned tool joints hardbanded with wear-resistant wire ARNCO 100XT by using the GMAW process led to the following observations:

- The parent metal (PM) structure is tempered sorbite (Figure 12a) that still keeps the martensite’s orientation (tempered martensite);
- The new hardbanding layers exhibits a typical weld deposit structure with alloys containing chemical elements that form carbides (Figure 12b);
- The transition area between the new hardbanding layers and parent metal presents a not-equilibrium phase (acicular martensite) in the parent metal without any discontinuities (Figure 12c);
- The adhesion between the new and old hardbanding layers exhibits an excellent interpenetration of the layers (Figure 12d), and a large amount of precipitated carbides was observed in the area of old hardbanding complete melted layers (Figure 12e);
- The heat-affected zone (HAZ) exhibits a coarse acicular martensitic structure typical of the area adjacent to the fusion line (Figure 12f);
- All the metallographic analyses performed on the reconditioned tool joint hardbanded with wear-resistant wire ARNCO 100XT have shown good adhesion, without any discontinuities or cracks, in the transition areas between new hardbanding layers/old hardbanding layers/parent metal/hardbanding layer overlaps.

**Figure 13:**
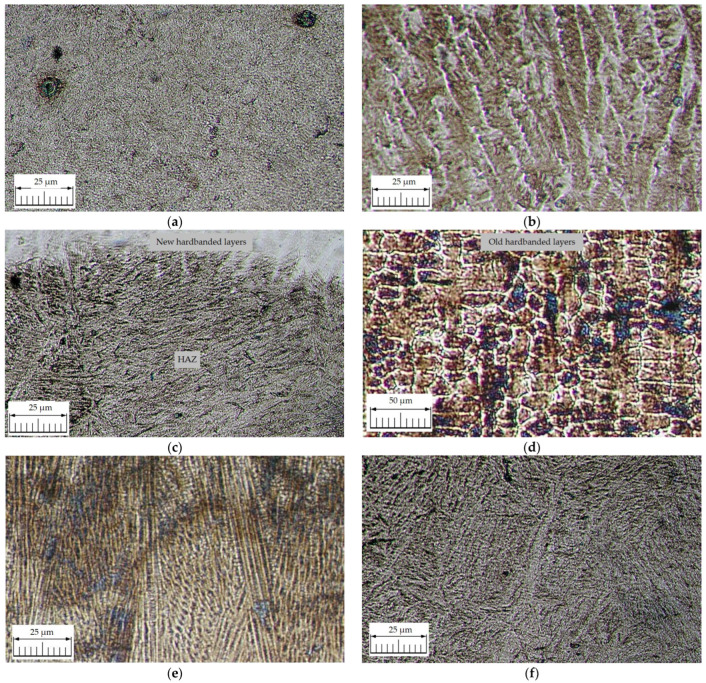
Images of metallographic structures for the reconditioned tool joints hardbanded by using the wear-resistant material FLUXOFIL M58 performed on sample B: (**a**) parent metal (HV0.1 = 341…258); (**b**) new hardbanding layers (HV0.1 = 553…504); (**c**) transition area between new hardbanding layers and the parent metal; (**d**) transition area between new and old hardbanding layers (HV0.1 = 642…540); (**e**) complete melting area of the old hardbanding layers (HV0.1 = 642…540); (**f**) heat-affected zone—HAZ (HV0.1 = 442…341).

Figure 13 features images from different areas of reconditioned tool joints that were hardbanded by using the wear-resistant material FLUXOFIL M58 (sample B) recorded during the metallographic analysis.

The metallographic investigations of the reconditioned tool joints hardbanded with wear-resistant wire FLUXOFIL M58 by using the GMAW process led to the following information:

- The structure of the parent metal is a very fine tempered sorbite (Figure 13a);
- The new hardbanding layers exhibit a typical weld deposit structure for alloys containing chemical elements which form carbides (Figure 13b);
- Investigations of the transition area between the new hardbanding layers and the parent metal (Figure 13c) revealed no discontinuities, with the parent metal exhibiting a non-equilibrium structure (acicular martensite);
- In the transition area between the new and the old hardbanding layers, a carbides network, due to the heating of the old hardbanding layers, was formed (Figure 13d) which was precipitated in large quantities, especially in the area of complete melting of the old hardbanding layers (Figure 13e); also, in this area, no defects were observed (submicroscopic voids, crack initiators);
- The metallographic constituent in the heat-affected zone is acicular martensite with course aspect, which is structure-specific to the area next to the fusion line (Figure 13f);
- All investigations showed good adhesion, with no discontinuities or cracks being seen in the transition areas between new hardbanding layers/old hardbanding layers/parent metal/hardbanding layer overlaps in the case of tool joints hardbanded with wear-resistant wire FLUXOFIL M58.

#### 3.1.3. Hardness Measurements

Hardness is a very important characteristic required to fulfill the quality requirements imposed on hardbanded tool joints during exploitation work to guarantee their protection against wear. In order to validate the quality of the tool joints hardbanded by using the technology developed during these experimental studies, the authors performed hardness measurements on metallographic samples A and B.

The Rockwell hardness (HRC) measurements were performed using the equipment type KRAUTKRAMER MIC 10 (manufactured by GE Inspection Technologies, Koln, Germany). According to the results presented in Table 3, the hardnesses of the new hardbanded areas are relatively constant throughout the thickness layers.

The high values of the hardness obtained due to the carbides formed after hardbanding of the tool joints with ARNCO 100XT (54.8…56.8 HRC) and FLUXOFIL M58 (54.6…55.2 HRC) are quite similar and fall within the hardness limits (52…57 HRC) imposed by operating conditions to ensure protection against abrasive wear. After the hardbanding process, the tempered sorbite presented in the parent metal and the hardness values measured (31.2…45.9 HRC) align with the characteristics of the tool joint quenched and high-tempered according to the API standards [3].

In addition, micro-hardness measurements were performed to investigate the quality of the hardbanded tool joints (Table 4).

The Vickers micro-hardness test was performed with the EMCO-DURASCAN 10 equipment (manufactured by EMCO-TEST, Kuchl, Austria), at a load of 100 g (HV0.1). The hardness measurements started from the hardbanded surface perpendicular to the parent metal, maintaining an indentation distance of 0.5 mm. According to the measurement indentations, these investigations also revealed a high level of hardness values in the new hardbanding area due to the carbide presences (797…626 HV0.1 for hardbanding with ARNCO 100XT and 553…504 HV0.1 for hardbanding with FLUXOFIL M58), which decrease through the heat-affected zone to the parent metal.

The tool joint parent metal remains unaffected by the hardbanding process, ensuring the integrity of drill pipes subjected to axial load during the exploitation work. The similarity between the new hardbanding layers and the old hardbanding layers regarding the hardness values and the structural characteristics has led to recommending the wear-resistant materials ARNCO 100XT and FLUXOFIL M58 for the reconditioning of used tool joints subjected to wear.

### 3.2. Welding Procedure Specification (WPS) for Hardbanding of the Tool Joints

**Figure 14:**
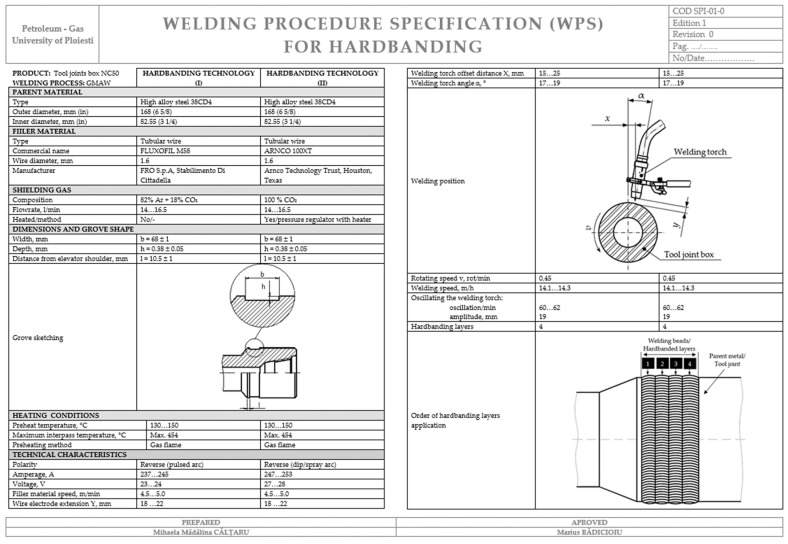
Welding procedure specification (WPS) for NC50 tool joints hardbanded with two wear-resistant materials ARNCO 100XT and FLUXOFIL M58 by using the GMAW process.

The quality control results validate the procedure that was documented in a welding procedure specification (WPS) to certify the quality of the technology developed for NC50 tool joints hardbanded with two wear-resistant materials, ARNCO 100XT and FLUXOFIL M58, using the GMAW process (Figure 14).

**Figure 15:**
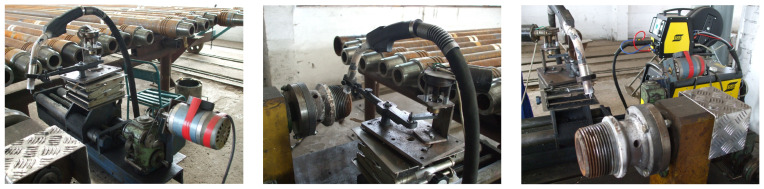
Images taken during the industrial process applied for hardbanding of the tool joints using the welding procedure specification developed within this research (Figure 14).

The technology, the equipment and the welding procedure specification (WPS) developed in the frame of this research are ready and adequate to be used in the reconditioning of tool joint drill pipes subjected to wear in industrial conditions (Figure 15).

## 4. Conclusions

The present research work established adequate reconditioning technology by hardbanding a real NC50 tool joint box with a 6 5/8 outer diameter, with the external surface having previously deteriorated due to the working conditions in the wellbore, conditions that also led to the wear of the hardbanding layers applied onto the external surface by the drill pipe’s manufacturer. The reconditioning process was performed by welding using the GMAW process and two different wear-resistant tubular wires (ARNCO 100 XT and FLUXOFIL M58) as filler material, in order to compare their applicability for re-hardbanding of the used tool joints. The wear-resistant materials are recommended by the manufacturers for use in the hardbanding of heavy worn parts. ARNCO 100 XT is recommended worldwide and used for the hardbanding of wear parts, especially in the petroleum industry [19,29,30,31,32].

The authors designed and manufactured necessary reconditioning equipment that was used to establish and apply the optimum hardbanding technological parameters. The hardbanding technology was developed by the authors in order to achieve the desired balance between the metallographic constitutes, structure integrity, hardness values and geometrical characteristics imposed by the working conditions of the hardbanded tool joints. The macroscopic analysis, the metallographic analysis by optical microscopy and the hardness measurements confirmed the quality of the hardbanding with ARNCO 100XT and FLUXOFIL M58. Both wear-resistant materials were adequate for use in the hardbanding of the tool joints. The macroscopic and microscopic analysis certified the quality of the deposited layers due to the fact that the hardbanded area contained no cracks, micro-cracks, voids or other discontinuities, highlighting the good penetration and good fusion between the new and the old hardbanded layers, between the new hardbanded layers and the parent metal and, also, between the two adjacent hardbanding layers. The hardness of the new hardbanding layers (52…57 HRC) of the reconditioned tool joint drill pipe align with the imposed exploitation conditions related to preventing the wear of the casing due to the friction process that may occur in the wellbore.

The results of the experimental research performed under laboratory conditions formed the basis for practical application of the process used for hardbanding and reconditioning of the NC50 tool joint box subjected to wear with two different wear-resistant materials named ARNCO 100X and FLUXOFIL M58, respectively. To ensure the reproducibility of the reconditioning technology, a welding procedure specification (WPS) was established, which is a mandatory document for the homologation process that constitutes the defining element for the qualification of operators and for ensuring the quality of hardbanding.

The authors have emphasized the importance of the studies conducted, highlighting that the research results serve as a guarantee of the validity and applicability of the recommended procedure in real situations specific to Oil Country Tubular Good (OCTG), which is used in the petroleum industry.

## Figures and Tables

**Table 1:** Deposited layers characteristics for wear-resistant materials.

Chemical Composition, %	FLUXOFIL M58	ARNCO 100XT
C	0.45–0.85	0.7-0.75
Mn	1.5–2.1	1 max.
Si	0.50–0.85	0.5–0.7 max.
P	0.020 max.	0.030 max.
S	0.020 max.	0.030 max.
Cr	5.0–7.0	7.0–8.0
Mo	0.35–0.85	0.15–0.20
Fe	Bal.	Bal.
**Hardness, HRC**	57–62	48–52

**Table 2:** Technological parameters of the hardbanding process by welding.

Technological Parameter	FLUXOFIL M58	ARNCO 100XT
Polarity	reverse	reverse
Average amperage I_s_, A	240	250
Average voltage U_a_, V	24	27
Shielding gas	82% Ar + 18% CO_2_	100% CO_2_
Shielding gas flow rate Q_g_, L/min	15	15
Filler material speed v_sm_, m/min	4.5	5
X distance (Figure 4b), mm	20	20
Y distance (Figure 4b), mm	21	21
Welding torch angle α (Figure 4b), °	18	18
Rotating speed of the specimen, rot/min	0.45	0.45
Welding speed v_s_, m/h	14.1	14.1
Oscillating speed of welding torch, oscillation/min	60	60
Oscillating amplitude of welding torch, mm	19	19
Preheat temperature T_pr_, °C	150	150
Maximum interpass temperature, °C	350	350

**Table 3:** Rockwell hardness values for reconditioned tool joints hardbanded with the wear-resistant tubular wire types ARNCO 100XT and FLUXOFIL M58.

Type of Wire Used for Tool Joint Hardbanding	Rockwell Hardness (HRC)		
	New Hardbanding Layers	Old Hardbanding Layers	Parent Metal
FLUXOFIL M58	54.6…55.2	52.8…53.3	31.2…45.9
ARNCO 100XT	54.8…56.8	54.2…55.7	40.8…44.9

**Table 4:** Vickers micro-hardness values for reconditioned tool joint hardbanded with the wear-resistant tubular wires ARNCO 100XT and FLUXOFIL M58.

Type of Wire Used for Tool Joint Hardbanding	Vickers Micro-Hardness (HV0.1)			
	New Hardbanding Layers	Old Hardbanding Layers	HAZ	Parent Metal
FLUXOFIL M58	553…504	642…540	442…341	341…258
ARNCO 100XT	797…626	626…516	595…390	390…285

## Data Availability

The original contributions presented in this study are included in the article. Further inquiries can be directed to the corresponding author.
